# Specific genes involved in synthesis and editing of heparan sulfate proteoglycans show altered expression patterns in breast cancer

**DOI:** 10.1186/1471-2407-13-24

**Published:** 2013-01-17

**Authors:** Iván Fernández-Vega, Olivia García, Ainara Crespo, Sonia Castañón, Primitiva Menéndez, Aurora Astudillo, Luis M Quirós

**Affiliations:** 1 Department of Pathology, Hospital Universitario Central de Asturias, Oviedo, 33006, Spain; 2 Department of Morphology and Cell Biology, University of Oviedo, Oviedo, 33006, Spain; 3 Department of Biotechnology, Neiker-Tecnalia Arkaute, Vitoria-Gasteiz, 01080, Spain; 4 University Institute of Oncology of Asturias, and Department of Functional Biology, University of Oviedo, Oviedo, 33006, Spain

**Keywords:** Heparan sulfate, Breast cancer, Proteoglycan, Glycosaminoglycan, Invasive ductal carcinoma

## Abstract

**Background:**

The expression of a specific set of genes controls the different structures of heparan sulfate proteoglycans (HSPGs), which are involved in the growth, invasion and metastatic properties of cancerous cells. The purpose of this study is to increase knowledge of HSPG alterations in breast cancer.

**Methods:**

Twenty-three infiltrating ductal adenocarcinomas (IDCs), both metastatic and non-metastatic were studied. A transcriptomic approach to the structure of heparan sulfate (HS) chains was used, employing qPCR to analyze both the expression of the enzymes involved in their biosynthesis and editing, as well as the proteoglycan core proteins. Since some of these proteoglycans can also carry chondroitin sulfate chains, we extended the study to include the genes involved in the biosynthesis of these glycosaminoglycans. Histochemical techniques were also used to analyze tissular expression of particular genes showing significant expression differences, of potential interest.

**Results:**

No significant change in transcription was detected in approximately 70% of analyzed genes. However, 13 demonstrated changes in both tumor types (40% showing more intense deregulation in the metastatic), while 5 genes showed changes only in non-metastatic tumors. Changes were related to 3 core proteins: overexpression of syndecan-1 and underexpression of glypican-3 and perlecan. HS synthesis was affected by lower levels of some 3-O-sulfotransferase transcripts, the expression of NDST4 and, only in non metastatic tumors, higher levels of extracellular sulfatases. Furthermore, the expression of chondroitin sulfate also was considerably affected, involving both the synthesis of the saccharidic chains and sulfations at all locations. However, the pro-metastatic enzyme heparanase did not exhibit significant changes in mRNA expression, although in metastatic tumors it appeared related to increased levels of the most stable form of mRNA. Finally, the expression of heparanase 2, which displays anti-metastatic features, experienced a strong deregulation in all patients analyzed.

**Conclusions:**

IDCs show alterations in the expression of HSPG genes; principally the expression and localization of proteoglycans and the sulfation patterns of glycosaminoglycan chains, depending on the metastatic nature of the tumor. In addition, the anti-proliferative molecule heparanase 2 experiences strong deregulation, thus highlighting it as a potentially interesting diagnostic factor.

## Background

Breast cancer is the most common form of cancer reported in women in all major regions of the world, comprising 22.9% of all non-melanoma skin cancers, and being responsible for 13.7% of cancer deaths in women [1]. The most frequent type of breast cancer is the invasive (or infiltrating) ductal carcinoma (IDC), which accounts for about 8 out of 10 invasive breast cancers. It starts in a duct, breaks through the wall of the duct, and invades the tissue of the breast, from where it is then able to metastasize to other parts of the body.

Invasive carcinoma cells characteristically induce changes in the adjacent stroma, and experimental evidence shows that the stroma in fact actively contributes to carcinoma progression [2]. Breast stroma accounts for more than 80% of resting breast volume and is composed of collagen, fibroblasts, endothelial cells, adipocytes and a molecular network of proteoglycans (PGs) [3]. The intralobular stroma, unlike the non-specialized interlobular stroma, forms a specialized functional unit with an abundance of PGs that facilitate hormonally-induced changes in breast volume and this constitutes the backdrop to the early stage of cancer invasion when the malignant transformation of tissue takes place.

PGs are a diverse group of glycoconjugates composed of different core proteins post-translationally modified with linear, anionic polysaccharides called glycosaminoglycans (GAGs) which consist of repeating disaccharides. Heparan sulfate proteoglycans (HSPGs) comprise a specific small group of proteins covalently linked to HS GAG chains. HS is a complex biopolymer initially created as a chain of alternating D-glucuronic acid (GlcA) and N-acetyl-D-glucosamine (GlcNAc). At various positions, the molecule is modified by a series of interdependent enzymatic reactions that include N-deacetylation of GlcNAc, usually followed by N-sulfation to produce GlcNSO_3_, thus creating sulfated S-domains. Within these regions, GlcA can be epimerized to iduronate (IdoA), and O-sulfate groups can be added at C6 of GlcN and C2 of IdoA residues. Minor sulfations at C3 of GlcN and C2 of GlcA may also occur. Chain modification results in clusters of flexible highly sulfated IdoA-rich regions, separated by more rigid lowly or non-sulfated regions [4,5]. HSPGs are ubiquitously present in tissues, mainly associated with the cell surface and the extracellular matrix (ECM) [4,5] and a variety of both normal and pathological functions have been ascribed to them, including cell adhesion and migration, organization of the ECM, regulation of proliferation, differentiation and morphogenesis, cytoskeleton organization, tissue repair, inflammation, vascularization and cancer metastasis [4-8], the function ultimately depending on the fine structure of the chains. Specific sets of variably modified disaccharides, usually within the sulfated domains, define binding sites for a multitude of specific ligands such as cytokines, chemokines, growth factors, enzymes and enzyme inhibitors, and ECM proteins [5,6].

Cells exercise exquisite control over HSPG composition and sequence, though this varies between cell types, development stages, and also as a result of cell transformation in pathological processes. It is therefore of interest to analyze in detail the complete set of changes in the expression of PGs and HS biosynthetic enzymes in cancer pathologies as well as the effect of these specific signatures on promoting invasion and metastasis.

In several cancer cells, genes involved in the biosynthesis of HSPGs are either up- or down- regulated. As such, the upregulation of two cell-surface PGs, glypican-1 (GPC1) and syndecan-1 (SDC1), and of the extracellular sulfatase Sulf-2, have been described in malignant breast cancer tissues [9,10] whilst the downregulation of some genes including *SULF1* and *HS3ST2* has also been reported [11-13]. However, to date no studies have analyzed the entire set of genes involved in the synthesis of these molecules in this pathology.

In this paper, investigation of HSPG’s biological function in IDCs was undertaken by analyzing the expression patterns of the genes involved in HSPG biosynthesis and comparing them with healthy tissues from the same patients. The tumors studied were subdivided into two groups according to presence or absence of metastases in lymph nodes since this element is a key predictor of progression. The study included genes coding for HSPG core protein and for enzymes responsible of HS chain synthesis and modification. Taking into account that some of these PGs can also carry chondroitin sulfate (CS) chains, we extended the study to the genes involved in the biosynthesis of this GAG. The aim of the work was to increase our knowledge of structural alterations of HSPGs in breast cancer, which could be of future benefit in the development of new chemical biology approaches to the retarding of tumor progression through the modulation of deregulated biosynthetic pathways.

## Methods

### Materials

The following materials were purchased from the manufacturers indicated: RNeasy Kit and RNase-Free DNase from Qiagen (Hilden, Germany); High-Capacity cDNA Reverse Transcription Kit and PowerSYBR Green PCR Master Mix from Applied Biosystems (Foster City, CA); GenElute PCR clean-up kit and 3-3^′^ diaminobenzidine from Sigma-Aldrich (St. Louis, MO); Biotin 3^′^ End DNA Labeling Kit from Thermo Scientific (Waltham, MA); In Situ Hybridization Detection System For Biotinylated Probes, EnVision™ G|2 Doublestain System and Envision FLEX target retrieval solution of high pH from Dako (Glostrup, Denmark);. All other chemicals were obtained from commercial sources and were of analytical grade.

The following antibodies were used in this study: Goat Anti-heparanase 1 (L-19), rabbit anti-sulf1 (H-81), rabbit anti-perlecan (H-300) and rabbit anti-chondroitin 6-sulfotransferase-2 (Z-24), all of which polyclonal antibodies were purchased from Santa Cruz Biotechnology, Inc (Santa Cruz, CA). Rabbit anti-heparanase-2 polyclonal antibody from GeneTex (Atlanta, GA), and mouse monoclonal anti-syndecan1 from DakoCytomation (Carpinteria, CA). Anti-mouse (sc-2020), anti-rabbit (sc-2004) and anti-goat (sc-2005) secondary antibodies were also from Santa Cruz Biotechnology (Santa Cruz, CA).

### Tissue samples

We analyzed a cohort of 46 snap frozen breast samples, obtained from the Tumor Bank at the Institute of Oncology of Asturias (Asturias, Spain). Twenty three of the samples were from IDCs while the remaining twenty three were from the corresponding surrounding healthy tissue from the same patients and were used as control. Diagnoses were evaluated using hematoxylin-eosin-stained slides of all samples according to the World Health Organization (WHO) criteria and the snap frozen tissues were stored at −80°C prior to isolation of the RNA. Applying the TNM classification, all tumors were at the T2 stage and were classified into two groups depending on the presence (at least N1) or absence (N0) of lymph node metastases, which resulted in 10 samples being included in the first group and 13 in the second. The study was approved by the Ethics Committee on Clinical Investigation of the Hospital Universitario Central de Asturias and all patients gave their consent.

### Total RNA isolation and cDNA synthesis

To obtain the RNA, fragments of tissue of between 20 and 30 mg in weight were used. Samples were homogenized using a polytron PT 2100 (Kinematica Inc; Bohemia, NY), and RNA was isolated using the RNeasy kit, following the manufacturer’s specifications. To ensure removal of residual contaminating DNA, samples were subjected to treatment with RNase-free DNase during the purification process itself. The concentration of RNA obtained was determined spectrophotometrically by measuring absorbance at 260 nm of a 1:50 dilution using a BioPhotometer (Eppendorf; Hamburg, Germany). The samples were divided into aliquots of 10 μl and used for reverse transcription reactions or stored at −20°C until further use.

cDNA synthesis was carried out using the High Capacity cDNA Transcription Kit following the manufacturer’s specifications. The reactions were performed using a thermocycler iCycler IQ (BioRad; Hercules, CA), using 2 μg of RNA as starting material. The reaction products were cleaned using the PCR Clean-Up GenElute kit following the manufacturer’s instructions. Finally, the aliquots containing the cDNA were diluted 1:20 with water and used for qRT-PCR assays or stored at −20°C until use.

### qRT-PCR reactions

In all cases, specific oligonucleotides were designed on different exons or exon junctions, using the program Primer 3. (http://biotools.umassmed.edu/bioapps/primer3_www.cgi). The size of the amplicon was situated in all cases between 70 and 150 base pairs, ensuring wherever possible that the Tm was above 77°C. The theoretical Tm for each amplicon was determined using the program Biomath (http://www.promega.com/biomath/calc11.). Primer sequences are presented in Additional file 1.

At least four repetitions of all the qRT-PCR reactions were carried out in a final volume of 10 μl, according to the manufacturer’s specifications, using 1 μl of the cDNA dilution as template, with 2 μl of primer pair mix (200 nM final concentration) and 5 μl of SYBR Green mix all assembled in 96 well microtiter plates. The plates were sealed with optical film and centrifuged at 2500 rpm for 5 min before being placed in a Real-Time ABI Prism Detection System device (Applied Biosystems; Foster City, CA) using the following cycling conditions: 95°C for 10 min, 40 cycles of 95°C for 15 s followed by 60°C for 60 s. Following the thermal cycling and data collection steps, amplimer products were analyzed using a melt curve program (95°C for 1 min, 55°C for 1 min, then increasing by 0.5°C per cycle for 80 cycles of 10 s each). For each amplification the presence of a single peak with a Tm corresponding to that previously calculated was verified. In those cases in which the amplifications were not adequate, new primer pairs were designed. Actin was included on each plate as a control gene to compare run variation and to normalize individual gene expression.

### Data analysis

To calculate the efficiencies of amplification for each gene we used the program LinRegPCR (http://www.gene-quantification.de/download.html), using the best correlation coefficient (considering a minimum of 3 points within the window of linearity) and establishing the average of all positive amplifications. At least 4 replicates of each reactions were carried out, with the number of replicates being increased in those reactions that showed ambiguity or dispersion of results. The values of differential expression of the genes of interest were expressed as has been described previously [14]. A non parametric Wilcoxon test was used for the statistical analysis of the experiments using a level of significance of p < 0.05. All analyses were performed using the Statistics for Windows program (Statsoft Inc; Tulsa, OK).

### Riboprobe preparation

Specific sense and antisense riboprobes for NDST4, SULF2 and HS3ST4 were designed. The riboprobe sequences were: NDST4 sense 5^′^- GCTGCTCCTGCTCTGCTGTTGCTAGTGCTGCTGTGC 3^′^, antisense 5^′^ GCACAGCAGCACTAGCAACAGCAGAGCAGGAGCAGxC 3^′;^ HS3OST4 sense 5^′^- TGTGGGGAGGGAGGAAGTCAGGGGTTGTGGGATGA 3^′,^ antisense 5^′^ TCATCCCACAACCCCTGACTTCCTCCCTCCCCACA 3^′;^ SULF2 sense 5^′^ CTCGCGCTCGCCTCCAGCCACACACATTTGCCATT 3^′,^ antisense 5^′^ AATGGCAAATGTGTGTGGCTGGAGGCGAGCGCGAG 3^′^. In all cases, the length of the probes was adjusted to between 34 and 36 nucleotides, the content of G + C to between 48% and 62% and Tm was always above 73°C. The probes were labeled with Biotin kit 3 ^′^End DNA Labeling Kit according to the manufacturer’s specifications.

### Chromogenic *in situ* hybridization (CISH)

To perform the hybridizations, tissue sections in paraffin were treated with xylene to render them diaphanous, the paraffin later being removed by passing it through decreasing alcohol concentrations until it water was reached. The samples were then incubated at pH 9 in buffer DAKO K8005 for 30 minutes at 90°C to facilitate the exposure of cellular ribonucleic acid. Subsequently, the preparations were washed with sterile tris-buffered saline (TBS), and incubated with labeled probes at a dilution of 1:2.5 in sterile water in a DAKO hybridization oven for 5 minutes at 95°C, followed by 15.5 hours at 62°C. Then, the preparations were washed with TBS for 10 minutes, followed by a second wash for a further 5 minutes. The entire procedure was carried out using the In Situ Hybridization Detection System for Biotinylated Probes according to the manufacturer’s specifications. Sections were fixed, mounted and examined with a Leica DMR microscope (Wetzlar,Germany). Visualization was carried out using a DFC295 Leica camera.

### Inmunohistochemistry

Tissue sections were dewaxed as described in the previous section. Rehydrated sections were rinsed in phosphate buffered saline (PBS) containing 1% tween-20. For detection of sulfatase-1, heparanase 2, chondroitin 6-sulfotransferase-2 and perlecan, sections were heated in high pH Envision FLEX target retrieval solution at 65°C for 20 min and then incubated for 20 min at room temperature in the same solution. For detection of heparanase the final step was omitted.

Endogenous peroxidase activity (3% H_2_O_2_) and non-specific binding (33% fetal calf serum) were blocked and the sections were incubated overnight at 4°C with primary antibodies using a 1:100 dilution. Secondary antibodies were used at a 1:100 dilution. 3-3’ diaminobenzidine was used as a chromogen. Selected slides were lightly counterstained with haematoxylin.

## Results

### Analysis of differential gene expression

We investigated the differential expression of the genes involved in defined steps of the biosynthesis of HSPGs in IDCs dividing the sample into two depending on the presence or absence of metastases in the lymph nodes.

13 samples were obtained from patients lacking metastases; their mean age was 60 ± 12 years; histological grade in all cases was moderate-low; the average tumor size was 2,08 ± 0,9 × 1,35 ± 0,6 cm; 90% were luminal A; 65% were located in the upper outer quadrant and 7.5% showed vascular invasion.

In addition, 10 samples were obtained from patients who showed lymph node metastases in 100% of cases, their mean age was 58 ± 14 years; histological grade was moderate-high; the average tumor size was 3,7 ± 0,4 × 3,11 ± 1,9 cm; The percentage of luminal A was 60%; only 20% were located in the upper, outer quadrant and 80% showed vascular invasion.

We used qRT-PCR to perform a quantitative analysis of mRNA expression. In many of the genes in which we were able to detect differences between normal tissues and tumors we complemented the studies determining the expression by histological techniques by including in situ hybridizations and Inmunohistochemistry.

### Differential expression of genes encoding core proteins carrying HS chains

**Figure 1:**
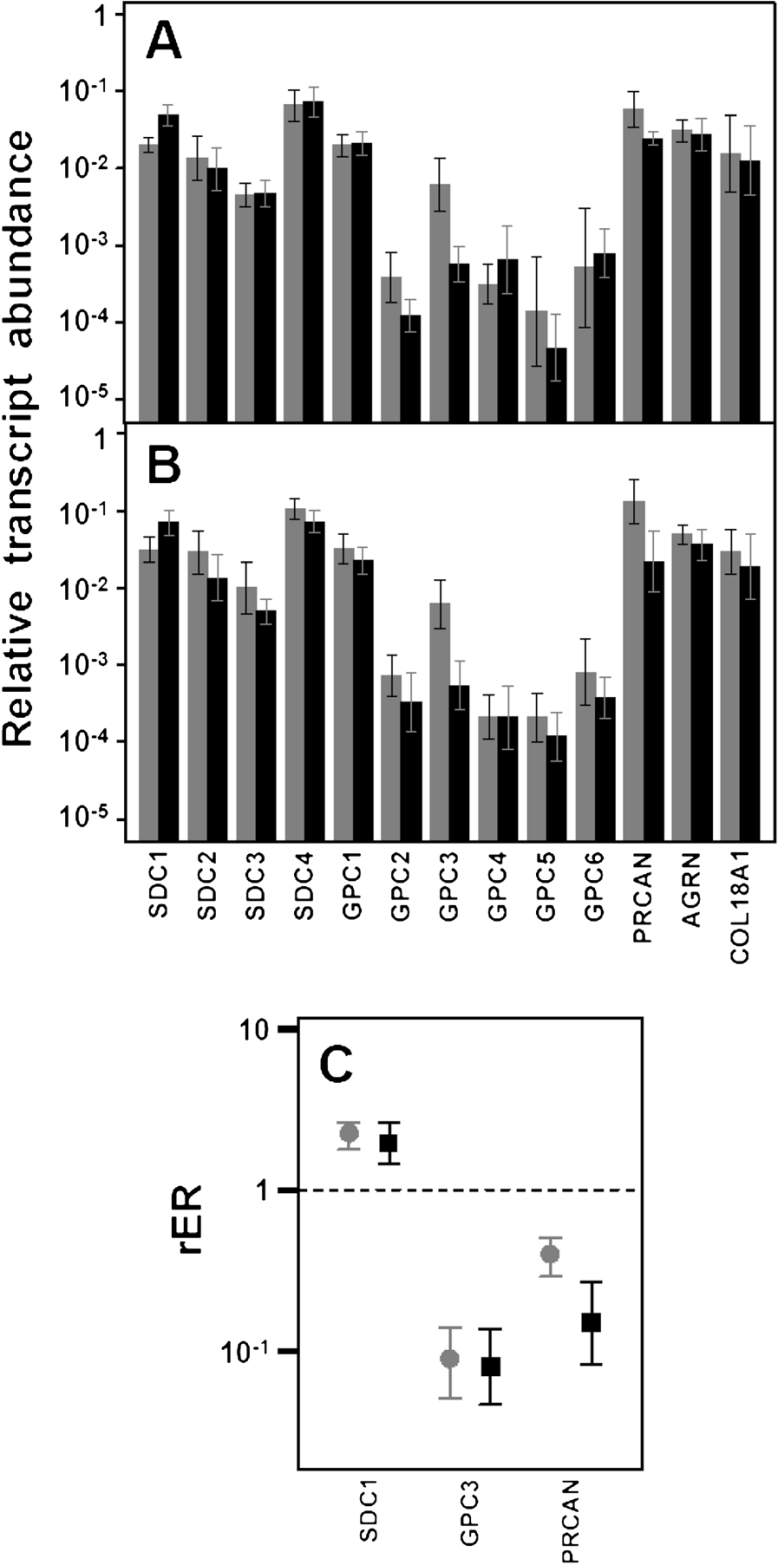
**Differential transcription of genes encoding HSPGs.** (**A,B**) Relative transcript abundance of mRNAs for HSPGs. Relative abundance for healthy tissues (gray bars) and tumors (black bars) are plotted on a log scale for each gene assayed and the spreads represent the standard deviations. (**A**) Non-metastatic IDCs. (**B**) Metastatic IDCs. (**C**) Relative expression ratio of genes that show statistically significant differences in expression in non-metastatic (●) or metastatic (■) IDCs. Values on the Y axis are represented on a logarithmic scale.

**Figure 2:**
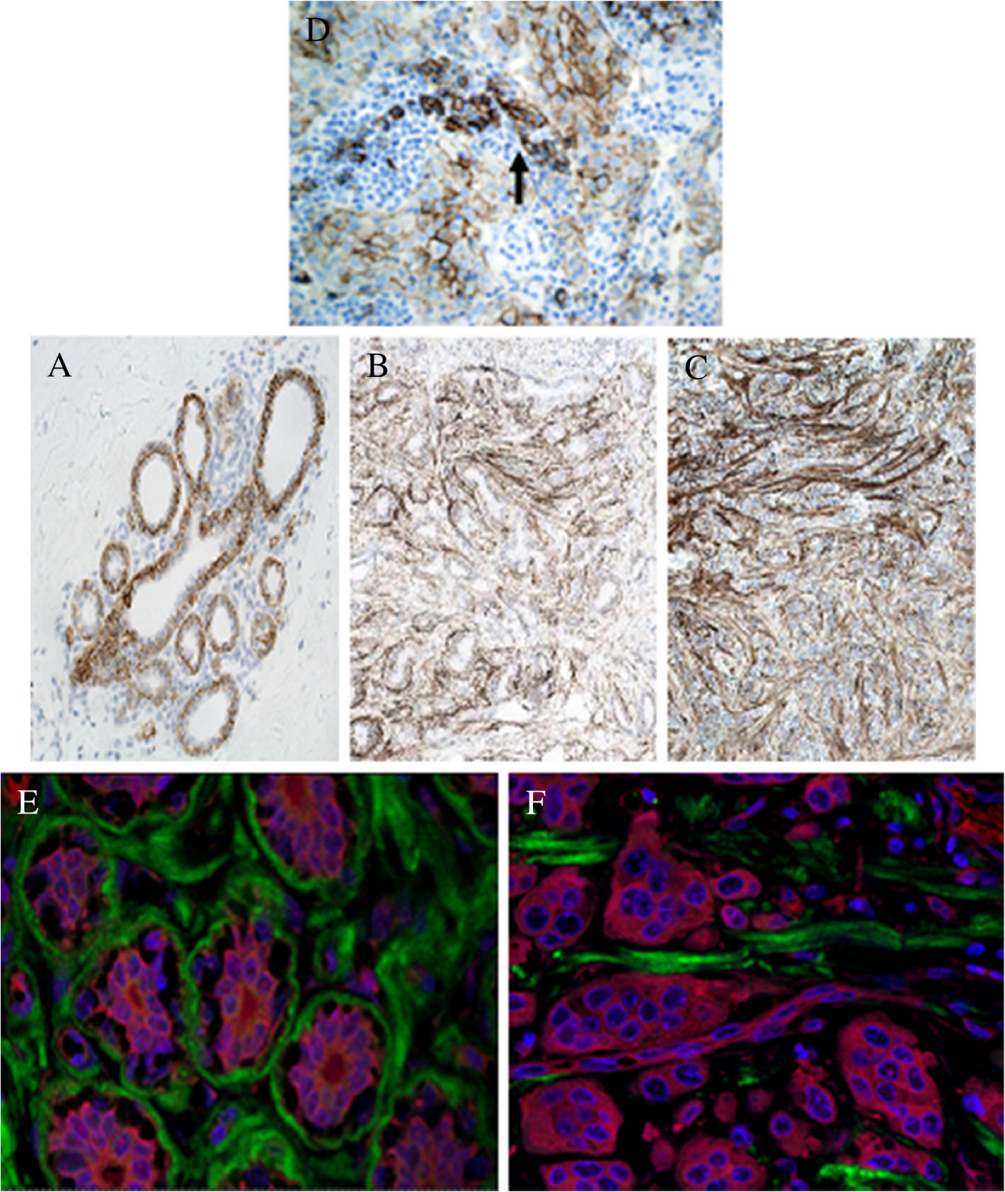
**Inmunohistochemistry of HSPGs.** (**A-C**) Localization of syndecan-1 using inmunhistochemistry in normal and tumoral breast tissue. (**A**) Normal tissues showing sydecan expression exclusively in the terminal ductal lobular unit, magnification 200X. (**B**,**C**) Non-metastatic and metastatic tumors, respectively,showing an increased expression of syndecan-1 in the desmoplastic stroma, magnification 100X. (**D**) Control of syndecan antibodies. IDC in area of chronic inflammatory reaction, where some plasmatic cells are present. They show strong positivity to syndecan (arrow). (**E**,**F**) Confocal microcopic visualization of perlecan (green) in IDC. The cytoplasm was stained with TriC-conjugated phalloidin (red) and nuclei counterstained with DAPI (blue). (**D**) Normal breast tissue. (**E**) Metastatic tumor tissue, magnification 600X.

Only 13 genes encode HSPG core proteins. Two gene families, syndecans and glypicans, account for most cell surface HSPGs. Respectively these families comprise 4 (*SDC1-4*) and 6 (*GPC1-6*) different proteins. The three remaining molecules are arranged in the extracellular matrix and include perlecan (*PRCAN*), agrin (*AGRN*) and collagen type XVIII (*COL18A1*) [15]. Within the syndecans group, no significant differences in the transcript levels of species 2, 3 and 4 could be detected (Figure 1A and 1B); however, Syndecan-1 displayed a more than two fold overexpression in both metastatic and non metastatic (p = 0.013 and 0.028 respectively) tumors (Figure 1C); overexpression occurred in 60% of the cases of non-metastatic IDCs analyzed and in 75% of the metastatic. Changes in Syndecan-1 were also evaluated immunohistochemically using monoclonal anti-SDC1. Healthy tissue analysis showed intensive staining on the baso-lateral surface of epithelial cells of ducts and acini in duct-lobular units, with local staining in myoepithelial cells (Figure 2A). Furthermore, IDCs displayed positive immunoreactivity on the basal side of ducts, as well as intense staining of the stroma, regardless of the nature of the tumor (Figure 2B and 2C).

Analysis of the expression levels of all the different Glypicans showed substantial differences between them, much wider than for the Syndecans, reaching up to nearly 3 orders of magnitude, with Glypican-1 being the most abundant species. The qRT-PCR results were unable to detect significant diferences in the levels of transcripts except for Glypican-3 (GPC3), in which 85% of non-metastatic (p = 0.003) and all metastatic tumors (p = 0.005) evidenced a strong (approximately 10 fold and 12 fold respectively) sub-expression (Figure 1C).

When evaluating the extracellular matrix PGs, no significant differences were detected for agrin and collagen XVIII. However, perlecan experienced significant down regulation of expression in 70% of non-metastatic (p = 0.002) and 90% of metastatic cases (p = 0.01). The values of reduced relative abundance of mRNA ranged from over 3 times more in non-metastatic IDCs to up to 6 times more in metastatic tumors (Figure 1C). In the same vein, immunofluorescence staining confirmed that expression of perlecan protein was reduced in tumor tissue compared to in the surrounding healthy tissue (Figure 2D and 2E).

### Expression of enzymes involved in the biosynthesis of HS chains

**Figure 3:**
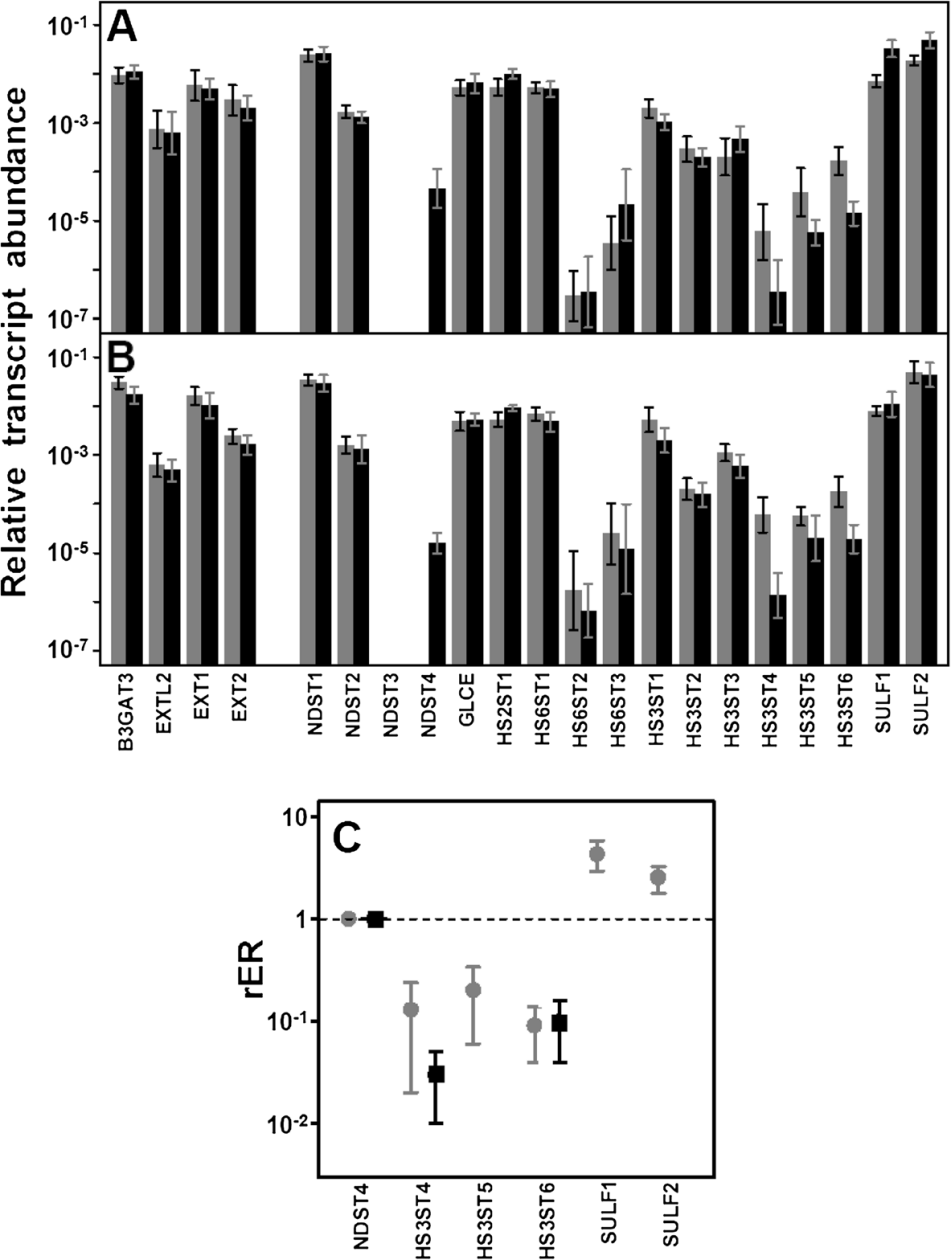
**Differential transcription of genes encoding enzymes involved in the biosynthesis of HS repeating unit.** (**A**,**B**) Relative transcript abundance of mRNAs for enzymes involved in the synthesis of HS chains. Relative abundance for healthy tissues (gray bars) and tumors (black bars) are plotted on a logarithmic scale for each gene assayed and spreads represent the standard deviations. (**A**) Non-metastatic IDCs. (**B**) Metastatic IDCs. (**C**) Relative expression ratio of genes that show statistically significant differences in expression in non-metastatic (●) or metastatic (■) IDCs. Values on the Y axis are represented on a logarithmic scale. NDST4 was not detected in healthy tissues.

HS and CS/DS chains are synthesized by cooperation of multiple biosynthetic enzymes in the Golgi. This study included the genes which code glycosyltransferases (GTs) involved in HS chain polymerization, including *EXTL2*, responsible for transferring the first GlcNAc residue, and *EXT1* and *EXT2*, which encode copolymerases for chain extension. None of the genes showed changes in their transcript levels in IDCs (Figure 3).

**Figure 4:**
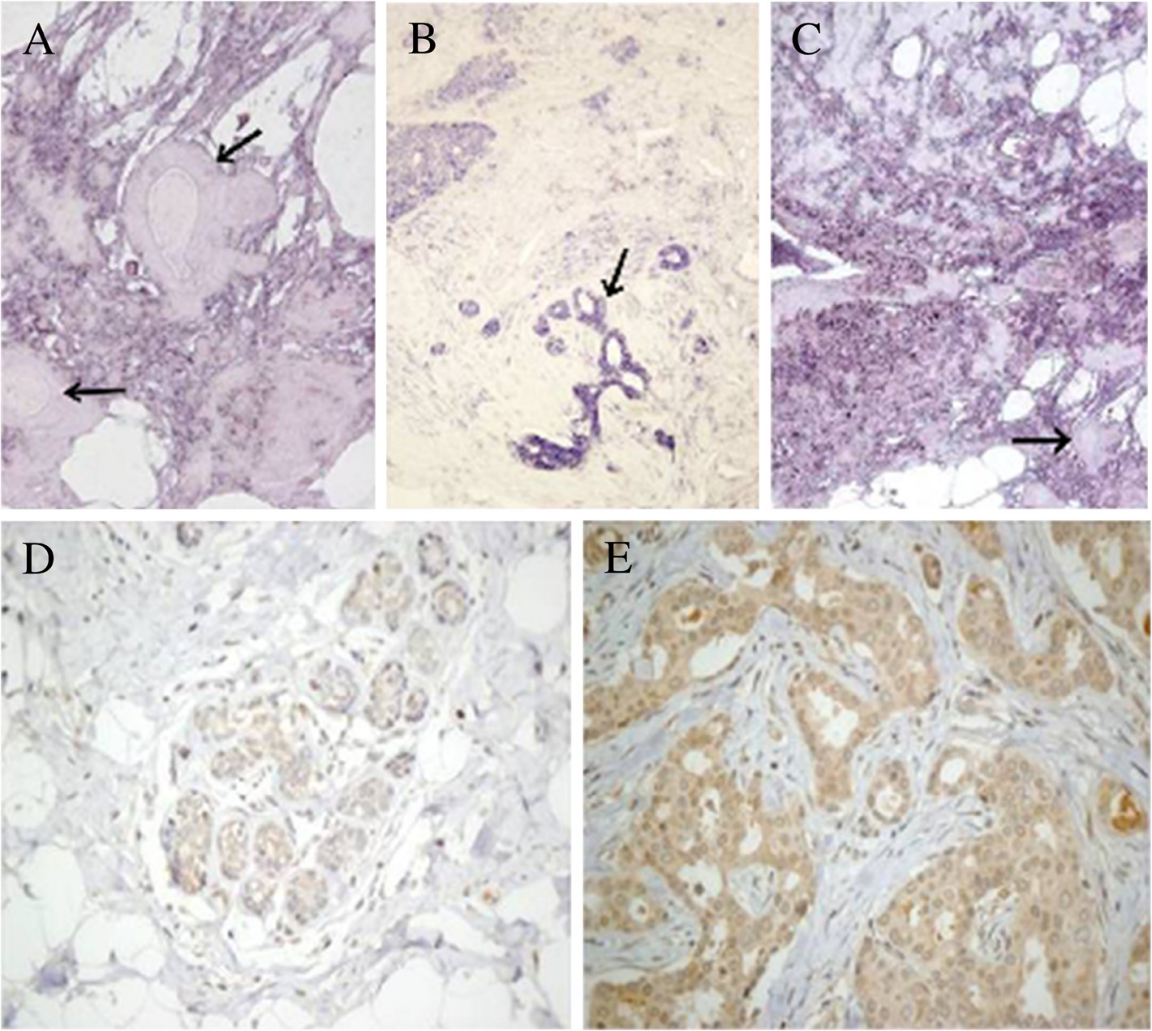
**Localization of enzymes involved in the modification of HS chains.** (**A**) CISH for NDST4 in IDC, negative for two ducts of normal appearance (arrows) and positive for the rest of the tissue, mainly consisting of tumor cells, magnification 200X. (**B**) CISH for HS3OST4 in IDC. The terminal ductal lobular unit showing an intense reactivity (arrow) while the staining patterns are less intense in tumor cells, magnification 100X. (**C**) CISH for SULF2 in IDC. At the bottom right unstained normal breast ducts are seen (arrow). Some adipose tissue is also present. Hybridization is positive for the rest of the tissue, constituted by tumor cells, magnification 100X. (**D**,**E**) Inmunohistochemistry for SULF1 in IDC. (**D**) Healthy tissue, terminal ductal lobular unit, magnification 200X (**E**) Intense immunostaining in tumor cells that grow into irregular ducts with lights, magnification 400X.

The result of the activity of all GTs involved in the synthesis of HS is an unmodified chain made of GlcA-GlcNAc repeating units. As the chain polymerizes, it undergoes several modifications; the initial ones involve removal of acetyl groups from GlcNAc residues, followed by sulfation of the amino group which is catalyzed by four different isoforms of N-deacetylase/N-sulfotransferases, NDST1, NDST2, NDST3 and NDST4 [4,16]. Transcripts of only two of these isoforms, NDST1 and 2, were able to be quantified in all healthy tissues, while NDST3 and 4 were undetectable in most patients (Figure 3A and 3B). When the analysis was conducted in tumor tissues, no significant differences of transcript levels of isoforms 1 and 2 were detected (Figure 3A and 3B). In addition, *NDST3* did not show clear alteration patterns, although its low expression did not allow for definitive conclusions to be drawn. Interestingly, *NDST4* transcription increased, and its expression was detected in 50% of patients, both metastatic and non metastatic (Figure 3). CISH studies confirmed this result, since tumors expressing *NDST4* showed a positive hybridization of tumoral cells that was absent in normal cells (Figure 4A).

Further modifications of the HS chain include the epimerization of GlcA into IdoA, catalyzed by the action of the enzyme C5-GlcA epimerase (*GLCE*), the addition of sulfate groups at C2 of uronic acid, catalyzed by the enzyme HS 2-O-sulfotransferase (*HS2ST1*), and the addition at C6 of glucosamine residues, catalyzed by HS 6-O-sulfotransferase isoforms 1–3 (HS6ST1, HS6ST2 and HS6ST3) [4,16]. None of these enzymes showed statistically significant alterations in transcriptional level (Figure 3A and 3B).

The last step in the modification of HS chains during biosynthesis in the Golgi involves the addition of sulfate group at C3 of glucosamine. This reaction is catalyzed by HS 3-O-sulfotransferase isoforms 1–6 (HS3ST1, HS3ST2, HS3ST3A1, HS3ST3B1, HS3ST4, HS3ST5 and HS3ST6) [17]. In non metastatic IDCs, isoforms 4, 5 and 6 exhibited an approximately 8, 5 and 10 fold downregulation respectively (p = 0.009, 0.01 and 0.01). Meantime, in metastatic tumors, while isoform 5 was not altered, there was a 10 fold reduction in expression of isoform 6 (p = 0.008), and isoform 4 down regulation (p = 0.02) increased more than 30 fold (Figure 3C). We carried out CISH studies with biotinylated probes for *HS3ST4* which confirmed a decrease in staining in the tumoral cells relative to normal tissue (Figure 4B).

The final modification of the HS patterning is carried out at the cell surface by two cell surface sulfatases, SULF1 and SULF2, which remove GlcN-6S groups from specific regions [17]. The transcript levels of both enzymes were overexpressed in non metastatic IDCs (p = 0.007 and 0.01 respectively, Figure 3). Interestingly, no significant differences could be detected in metastatic tumors. Inmunostaining and CISH techniques applied for SULF1 and SULF2 respectively corroborated this data, showing stronger staining in tumor cells relative to healthy ones (Figure 4C and 4D,E).

### Expression of enzymes involved in the biosynthesis of CS chains

**Figure 5:**
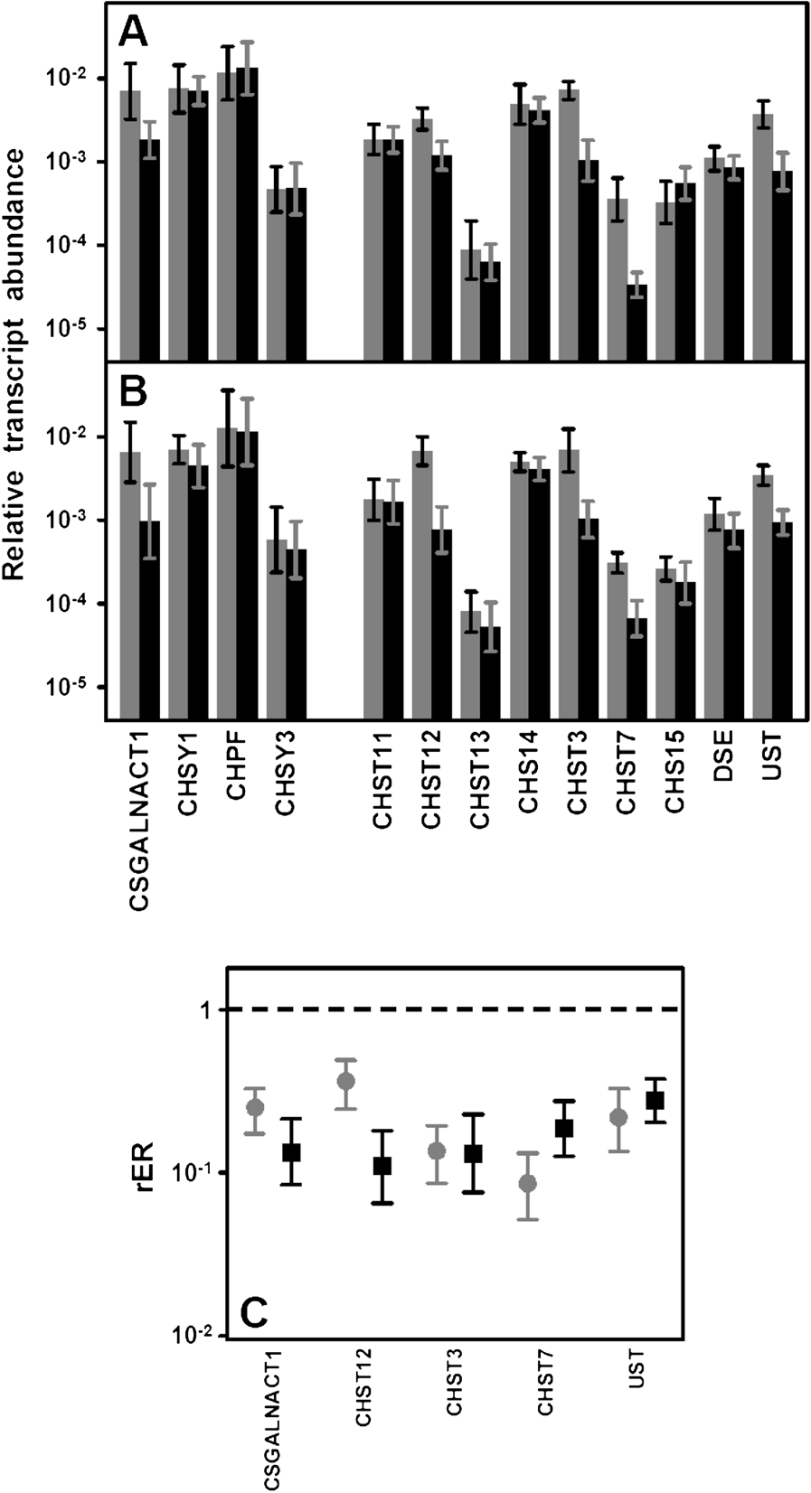
**Differential transcription of genes encoding enzymes involved in the biosynthesis of CS repeating unit.** (**A**,**B**) Relative transcript abundance of mRNAs for enzymes involved in the modification of CS chains. Relative abundance for healthy tissues (gray bars) and tumors (black bars) are plotted on a logarathmic scale for each gene assayed and spreads represent the standard deviations. (**A**) Non-metastatic IDCs. (**B**) Metastatic IDCs. (**C**) Relative expression ratio of genes that show statistically significant differences in expression in non-metastatic (●) and metastatic (■) IDCs. Values on the Y axis are represented on a logarithmic scale.

We analyzed the transcription levels of genes involved in the polymerization of CS chains, including *CSGALNACT2*, responsible for transferring the first N-acetyl-galactosamine (GalNAc) residue, and the chondroitin synthases *CHSY1*, *CHPF* and *CHSY3*[17]. With one exception, none of these genes showed changes in their transcript levels in either non-metastatic or metastatic IDCs (Figure 5A and 5B). The exception was *CSGALNACT2*, which was downregulated approximately 4 and 7 fold in non-metastatic (p = 0.01) and metastatic (p = 0.02) IDCs respectively (Figure 5C).

CS chains are modified to a lesser extent than those of HS. Possible reactions include epimerization of GlcA in CS chains, which results in dermatan sulfate (DS) chains, catalyzed by DSE; addition of sulfate groups at C2 of IdoA residue of DS, catalyzed by chondroitin uronosyl sulfotransferase (UST); sulfation at C4 of GalNAc, catalyzed by different isoenzymes with specificity for CS or DS chains (CHS11, CHS12, CHS13, CHS14), and addition of sulfate at C6 of GalNAc, also catalyzed by different isoenzymes (CHS3, CHS7); sulfation at C6 may also occur in pre-sulfated residues catalyzed by a N-acetylgalactosamine 4-sulfate 6-O-sulfotransferase (CHS15) [18].

**Figure 6:**
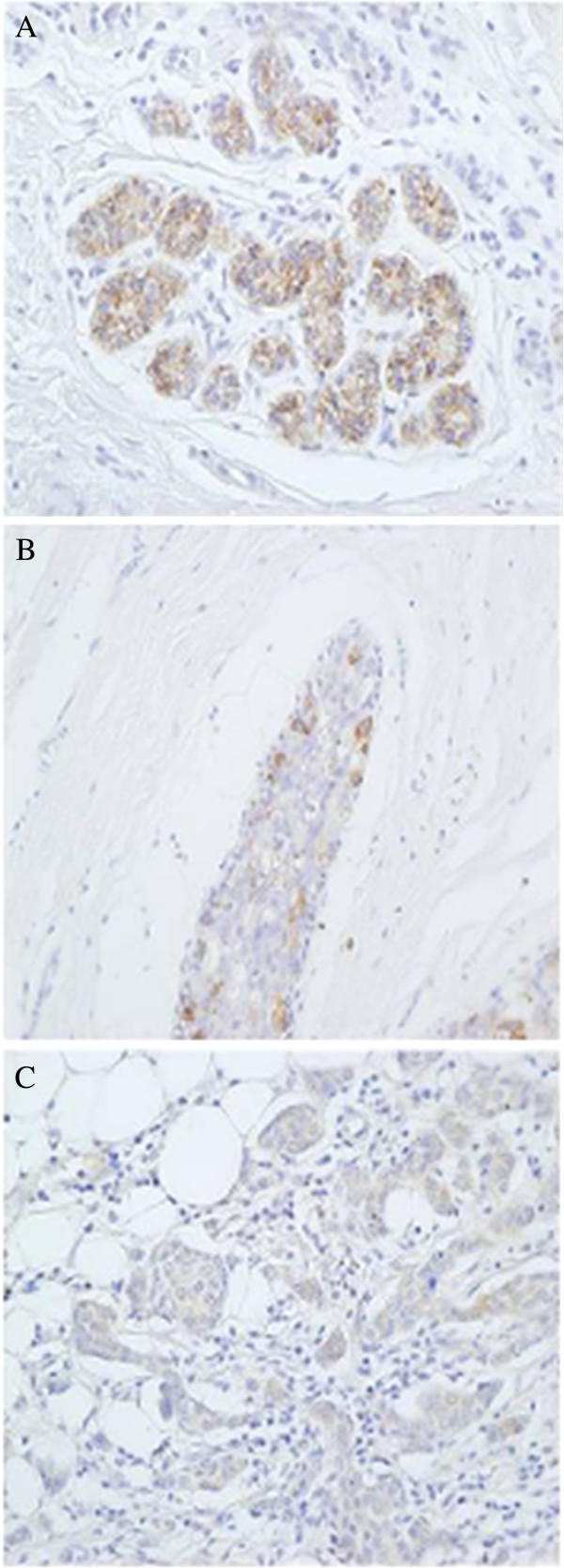
**Inmunolocalization of chondroitin 6-O-sulfotransferase 1.** (**A**) Typical terminal ductal lobular unit of the breast displaying an intense cytoplasmic immunoreactivity for CHST3. Perilobulillar inflammatory cells are not marked. (**B**) Breast duct with IDC showing cytoplasmic immunoreactivity only in cells of the basal layer. (**C**) Group of epithelial cells that proliferate haphazardly, infiltrating the adjacent adipose tissue. It is a IDC in which there is little immunoreactivity for CHST3, magnification 400X.

No changes affecting the epimerization of GlcA could be detected, but several reduced relative abundances of mRNA involving genes responsible for the sulfation at different locations were. C2 of IdoA residues seemed to be undersulfated since *UST* transcription decreased 4 fold both in non metastatic (p = 0.01) and metastatic (p = 0.01) IDCs (Figure 5). Sulfation at C4 of GalNAc appeared to be selectively affected since one of the chondroitin 4-O-sulfotransferases, *CHS12*, decreased 3 fold in non metastatic tumors (p = 0.02) and around 10 fold (p = 0.009) in metastatic IDCs (Figure 5). However, what did appear to be affected in a generalized form was the generation of GalNAc(6S) residues given that the transcription of the two genes coding the enzymes that catalyze this reaction, *CHST3* and *CHST7*, decreased in both non metastatic (p = 0.003 and 0.003 respectively) and metastatic (p = 0.009 and 0.01) IDCs (Figure 5). Inmunohistochemical studies were performed for CHST3 using specific antibodies; these analyses allowed us to visualize the decrease in immunostaining as tumor cell proliferate in relation to healthy tissue (Figure 6A-C).

### Expression of heparanases

**Figure 7:**
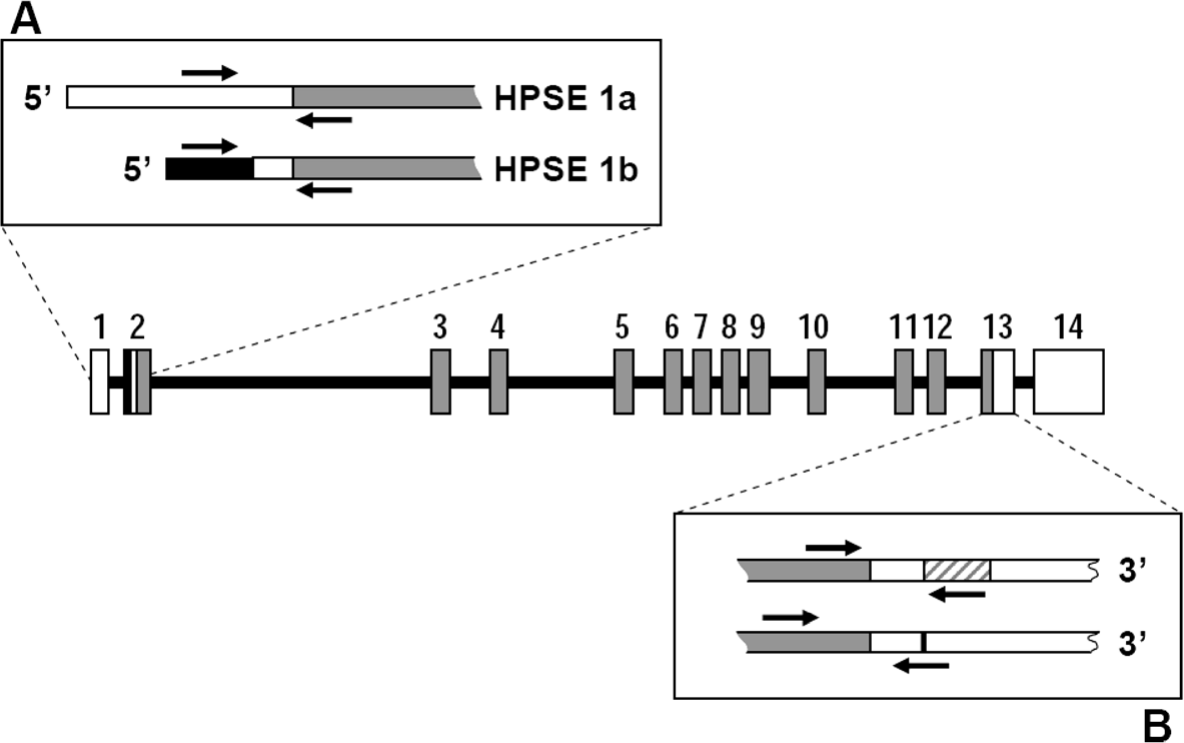
**Genomic organization of *HPSE *gene.** Numbered boxes represent the 14 exons. Gray boxes represent the ORF and white boxes represent the 5^′^ and 3^′^ untranslated regions. The black region represents the sequence of intron that form part of *HPSE 1b*. The location of PCR primers is indicated by arrows. (**A**) 5^′^ end of isoforms *HPSE1a* and *HPSE 1b*. (**B**) Location of the 185-bp sequence within the 3^′^ unstranslated region that mediates HPSE down-regulation.

**Figure 8:**
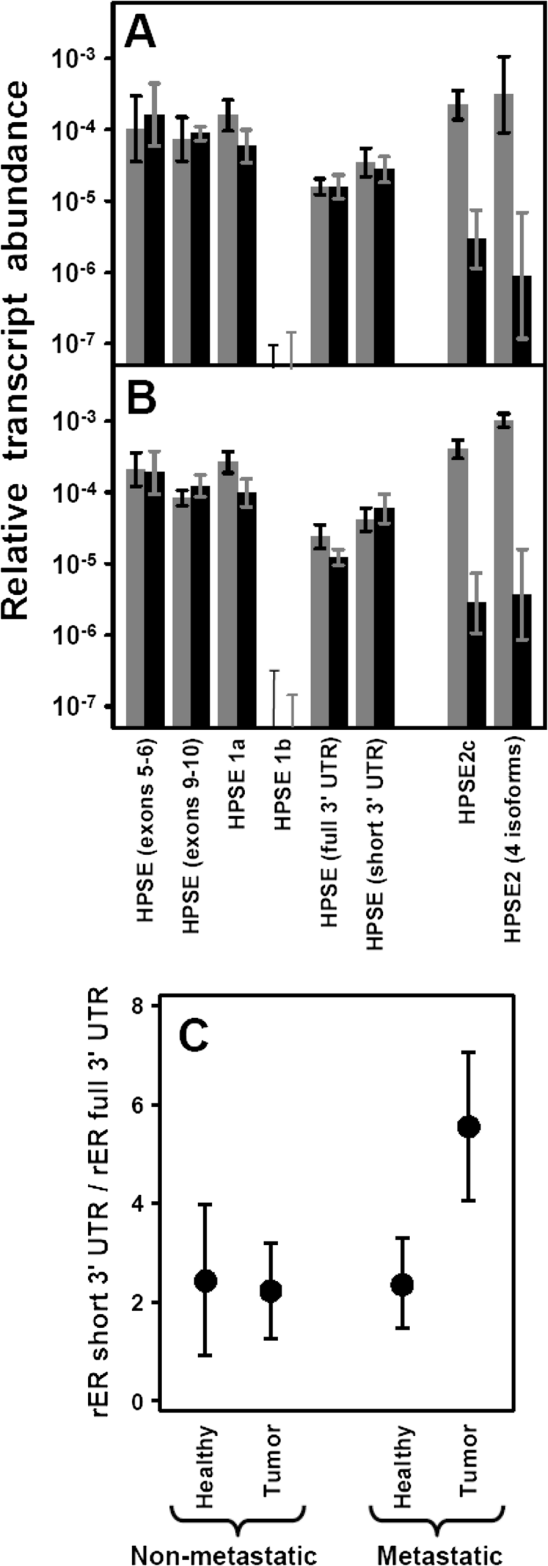
**Differential transcription of genes encoding heparanases.** (**A**,**B**) Relative transcript abundance of mRNAs for HPSE and HPSE2 isoforms. Relative abundance for healthy tissues (gray bars) and tumors (black bars) are plotted on a logarithmic scale for each gene assayed and spreads represent the standard deviations. (**A**) Non-metastatic IDCs. (**B**) Metastatic IDCs. (**C**) Relative transcript abundance of 3^′^ UTR isoforms of heparanase in healthy tissues and non-metastatic and metastatic IDCs plotted on a linear scale.

Heparanase (HPSE) is an endo-β-D-glucuronidase that degrades HS, generating biologically active fragments. This enzyme, along with the previously mentioned SULF1 and SULF2, constitutes what are known as the editing enzymes, responsible for modification of HS fine structure in physiological and pathological processes. We analyzed its transcription in relation to the presence or absence of metastasis in IDCs. The structure of the HPSE gene is represented in Figure 7; it spans over 50 kb and consists of 14 exons [19]. Performing qRT-PCR reactions, we failed to detect significant changes in *HPSE* transcription either in metastatic or non metastatic IDCs (Figure 8), thereby apparently disagreeing with previous reports indicating overexpression of this enzyme, especially in metastatic tumors [20]. Our initial experiments were carried out using primers placed in exons 5 and 6 respectively; we also performed new reactions using primers located in exons 9 and 10, with similar results (Figure 8).

Possible alterations in the noncoding regions of mRNA that could influence the expression levels were also analyzed. Two mRNA species containing the same ORF, *HPSE1a*, and *HPSE1b*, generated by alternative splicing, have been described [19]. Both species differ in the structure at their 5^′^ end, which helps to differentiate them using appropriate primers (Figure 7A). qRT-PCR reactions indicated that the isoform HPSE1a was expressed in both healthy and tumor tissues, while HPSE1b remained at very low or undetectable levels (Figure 8A and 8B).

**Figure 9:**
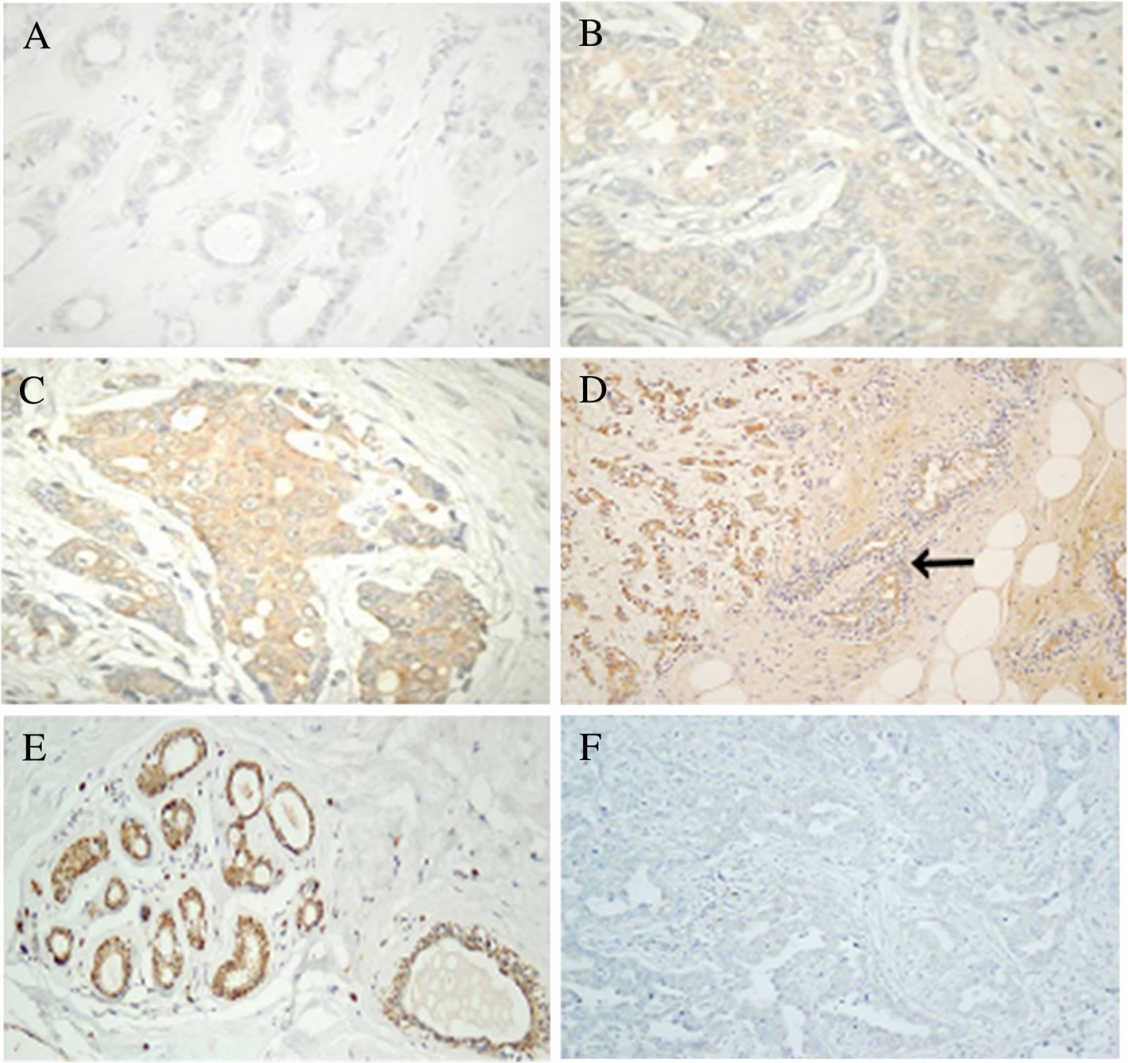
**Inmunohistochemistry of heparanases.** (**A-D**) Immunohistochemistry of HPSE. (**A-D**). Series of images showing the heterogeneous patterns of expression of HPSE in IDCs from negative staining (**A**), weak staining (**B**), moderate staining (**C**) and strong staining (**D**). Weak staining of breast normal tissue is indicated by an arrow (**D**); magnification A-C 400X, D 100X. (**E**,**F**) Immunohistochemistry of HPSE2. (**E**) Image of at least three terminal ductal lobular unit displaying immunostaining, mainly in the myoepithelial cells of ducts and acini. A slight reactivity in acinar and ductal cells can also be detected. (**F**) Tumor tissue forming ducts of different sizes and irregular light: The immunoreactivity for the antibody against HPSE2 is very low, magnification 200X.

Recent research has identified the existence of a 185-bp sequence within the 3^′^ untranslated region that mediates HPSE down-regulation [21]. We designed probes to detect the presence of this alteration in the 3^′^ end (Figure 7B). The results indicated that both forms could be detected, although the shorter species was the most abundant in all cases (more than two fold, Figure 8A and 8B). Comparison of expression levels of each of the transcripts did not evidence any significant differences between tumors and healthy tissue. However, when the relative expression levels of both forms (short form / long form expression ratio) were subjected to statistical analysis, there was indeed no significant difference in non-metastatic tumors, but there was in fact a change in metastatic relative to healthy tissues (*p* = 0.02), suggesting a higher relative proportion of the short isoform in this group of IDCs (Figure 8C). Alterations in the expression of HPSE were also determined in tissue arrays by immunohistochemistry and demonstrated the existence of varying levels of protein overexpression in different patients (Figure 9A-D).

Heparanase 2 (HPSE2) is a homologue of HPSE that lacks HS-degrading activity, although it is still able to interact with HS with high affinity [20]. Using a pair of probes designed to anneal to common regions in all isoforms predicted for this gene, we were able to detect a significant transcription alteration which appeared down-regulated approximately 30 fold (p = 0.01 and 0.007) in all types of IDCs (Figure 8A and 8B). It has been previously described that wild-type heparanase 2 (HPSE2c) exhibits very high affinity for HS, and is able to compete with HPSE. To check the expression levels of this isoform, we performed qRT-PCR reactions using probes against exons 3 (absent in isoform HPSE2b) and 4 (absent in isoforms HPSE2b and HPSE2a). The results were again of note, indicating as they did a 30 fold decrease in the expression of (p = 0.01) in IDCs (Figure 8A and 8B). Immunohistochemical studies confirmed this result, with tissues from all patients analyzed showing a decrease in immunoreactivity for antibodies specific for HPSE2 (Figure 9E and 9F).

## Discussion

**Figure 10:**
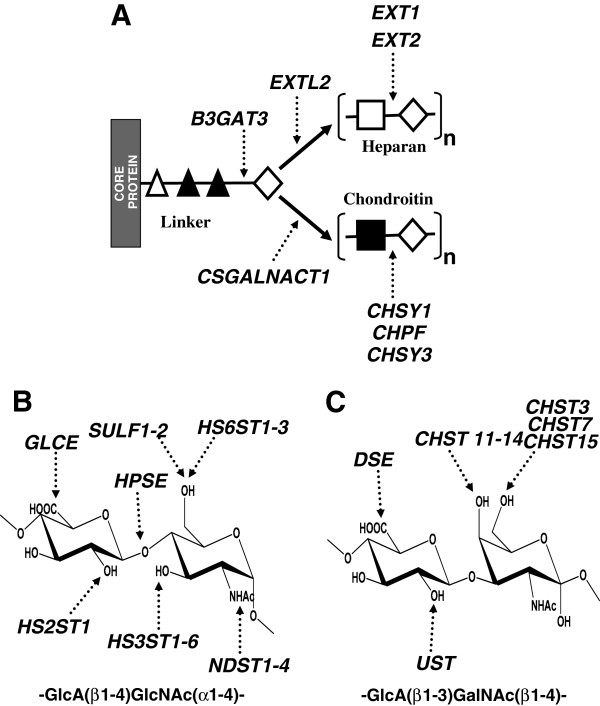
**Biosynthesis of heparan sulfate and chondroitin sulfate structures.** (**A**) GTs involved in HS and CS biosynthesis. (△) xylose; (▲) galactose; (♢) GlcA; (□) GlcNAc (■) GalNAc. (**B**) Modification genes involved in HS biosynthesis and edition. (**C**) Modification genes involved in CS biosynthesis.

The expression of HSPGs is markedly altered during malignant transformation and tumor progression, affecting both the PG core proteins and the GAG chains [13]. The HS fine structure can be determined by cell-type specific expression of only certain isoforms of some biosynthetic enzymes (Figure 10), notwithstanding the existence in some specific cases of regulation at translation level or enzymatic catalysis [21-24]. In this paper we investigated the expression patterns of the genes involved in HSPG biosynthesis in IDCs compared to those of healthy tissues from the same patients. The tumors were subdivided into two groups according to presence or absence of metastases in the lymph nodes, given this is the principal indicator of cancer progression.

In human cells, there are 13 genes encoding “full-time” HSPGs, although a few more may appear as “part-time” ones [4,5]. Only SDC1 from the syndecan group appeared overexpressed more than two fold in both metastatic and non metastatic tumors. Upregulation of SDC1 has been previously described in breast cancer, although with values exceeding 10 times those of the normal tissue, as determined by immunohistochemical staining quantification [25]. Our analysis of tumor and healthy tissue sections using monoclonal anti-SDC1 displayed an intense Immunoreactivity of the tumoral stroma regardless of the nature of the tumor, although this was more intense in metastatic IDCs, and lesser staining of the basement membrane of ducts.

This result represents a change in the location of the expression of SDC1 compared to healthy tissue, where immunoreactivity was displayed mostly on the baso-lateral surface of epithelial cells of ducts and acini in duct-lobular units. The shift of SDC1 from epithelial to stromal cells during progression of breast tumors has been previously reported, and suggested to stimulate carcinoma growth and angiogenesis [26,27]. Moreover, the existence of differences between transcript levels quantified by qRT-PCR and immunostaining found in the present study could be an indicator of additional post-transcriptional regulation of SDC1 expression, which has been described for certain cell types [24]. Upregulation of SDC1 has also been described in other tumors such as pancreatic, lung and brain cancer, and it has been postulated that this aberrant expression may play a key role in promoting growth factor signaling in cancer cells [13]. Interestingly, SDC1 is downregulated in various malignances, such as colorectal cancer, indicating that this HSPG may serve as a prognostic marker in a cancer-type-specific manner [13].

No significant differences in the levels of transcripts of isoforms 2, 3 and 4 were detected in this study, although overexpression of SDC4 has been previously described for an estrogen receptor-negative highly proliferative breast carcinoma subtype [28]; however, most samples analyzed in this study displayed a luminal A phenotype, which constitutes a common early stage of breast cancer where tumors have higher levels of estrogen and progesterone receptors.

Glypicans influence tumor progression and their expression is abnormal in various human tumors [22]. The analysis of the expression of the 6 protein isoforms in tumors only showed significant differences for GPC3. *GPC3* transcription was downregulated in 85% of non-metastatic and 100% of metastacic IDCs, with its levels decreasing about 10 and 12 fold respectively. Previous reports have detected similar results [22,29], comparable with those found for other tumor types, including lung, gastric, ovarian cancer and mesothelioma [22]. GPC3 plays a negative role in cell proliferation, and its depletion may contribute to cancer progression, although results for tumors originating from tissues expressing GPC3 in the embryo only, suggest its expression tends to occur together with malignant transformation [22].

Of the three extracellular matrix proteins, only perlecan evidenced a significant alteration of its transcript levels being downregulated 3 fold in non-metastatic IDCs and close to 6 fold in metastatic tumors. Perlecan is a critical regulator of growth factor-mediated signaling and angiogenesis, and is fundamental for the maintenance of basement membrane homeostasis [30], and as such its alteration could play important roles in IDC progression. Although expression of perlecan is enhanced in a number of tumor types, its levels are undetectable in some other instances such as lung carcinoma and in hepatocellular carcinoma cells [31]; in these latter cases, lack of perlecan has been suggested to perhaps favour the diffusion of growth factors, leading to tumor growth and metastasis [31].

Most, if not all, HSPGs can be hybrid molecules, carrying both HS and CS side chains [15]. To generate GAG chains requires the regulated expression and action of multiple GTs, which are arrayed in the lumen of the Golgi apparatus [4] (Figure 10A). Analysis of differential transcription of polymerases involved in the synthesis of HS chains (*EXTL2*, *EXT1*, *EXT2*) and CS chains (*CSGALNACT1*, *CHSY1*, *CHPF*, *CHSY3*) did not show any significant difference except for *CSGALNACT1*, which was down regulated arround 3 fold in both groups of IDCs. This gene encodes a chondroitin β1,4 N-acetylgalactosaminyltransferase which acts immediately after synthesis of the linkage tetrasaccharide and directs the synthesis towards the polymerization of CS chains. Competition exists between this reaction and the addition of a α1,4-linked GlcNAc, that directs the synthesis towards the polymerization of HS chains, such that the decline observed in this study would suggest a decrease of CS in relation to HS. Variations in the levels of GAGs in different tumors have previously been described, both increases and decreases. Also, in certain types of breast tumors, such as pericanalicular fibroadenoma, low levels of GAGs have been observed, particularly CS, while in intracanalicular fibroadenoma or scirrhous carcinoma the CS content increases but, interestingly, in no case have alterations in the levels of HS been detected [32]. The relative levels of galactosaminoglycans in these tumors have been suggested to have a close relationship with the fibrogenesis in the interstitial stromal elements [32].

HS fine structure depends on the expression and action of multiple sulfotransferases and an epimerase (Figure 10B). The initial modification reaction involves removal of acetyl groups from GlcNAc residues, followed by sulfation of the amino group catalyzed by four different isoforms of N-deacetylase/N-sulfotransferases [4,16], a reaction essential for the creation of sulfated S-domains. NDST1 and NDST2 show broad overlapping tissue distribution [18], have both been detected in breast tissue, but our results showed no significant differences of transcript levels in IDCs. NDST3 and NDST4, on the contrary, are expressed primarily during embryonic development [33]. In the present study neither of these isoforms were detected in healthy tissues; Furthermore, *NDST3* was only detected at very low levels and only in a small number of patients, making it difficult to draw conclusions. *NDST4* transcripts were, however, detected in 50% of tumors, both metastatic and non metastatic, a finding confirmed by subsequent CISH studies which selectively detected the presence of *NDST4* transcripts in tumoral cells and not in normal cells. NDST4 has been described as possessing different enzymatic properties to those of NDST1 and NDST2, in so far as it displays weak deacetylase activity but high sulfotransferase [34].

Further modifications of HS chains include the action of C5-GlcA epimerase and 2-O-sulfotransferase [5], and the sulfation at C6 of GlcN residues, catalyzed by HS 6-O-sulfotransferases [35]. There were no significant differences in tumor expression for any of these in the present work.

The last family of enzymes involved in HS modification are the 3-O-sulfotransferases, which add a sulfate group to C3 of already sulfated glucosamine residues [5]. In several cancers, including breast, a *HS3ST2* gene silenced via methylation has been reported [12,36]; however, in this study, the transcription of this gene was only downregulated in about 45% of cases analyzed. Nevertheless, *HS3ST6* displayed significant differences, appearing to be downregulated about 10 fold in more than 80% of metastatic and non-metastatic IDCs. *HS3ST4* was the isoform that showed the lowest transcription levels, confirming previous data showing low levels of expression of this isoform in most tissues, except in cerebral cortex and cerebellum [37]. Its transcription also displayed significant differences, exhibiting an 8 fold under-expression in 75% of metastatic tumors, and of more than 30 fold in 90% of metastatic. Finally, *HS3ST5* also appeared to be expressed only at low levels, again confirming previous findings of its low expression in all tissues [37]. Its transcript levels also displayed significant decreases, although only in the group of non-metastatic IDCs. Whilst the implications of 3-O-sulfation decrease in tumors are not yet known, it has been suggested that certain patterns of 3-O-sulfation could impart cancerous phenotypic changes [13].

Of the different sulfate groups present on the HS chains, 6S modification is the only sulfate moiety known to be post-synthetically edited from the chain [38], by a set of HS 6-O-endosulfatases localized in the cell surface, SULF1 and SULF2. The analysis of both genes in IDCs showed significant differences in non-metastatic tumors, in which both appeared overexpressed in 70% of the cases analyzed. By contrast, in metastatic tumors the percentage decreased to 40%. Overexpression of these genes has been previously reported in other tumors such as pancreatic cancer [13]. With regard to breast cancer, previous studies indicate an upregulation of *SULF2*[10], while *SULF1* has been found to be down regulated in some breast cancer cell lines [11], although in our study we could only detect a small reduced relative abundance of mRNA of SULF1 in less than 10% of the cases analyzed. The alterations in sulfation patterns in tumors have been suggested to perhaps be related with protecting the cancer cell from Natural Killer (NK) recognition [13].

As indicated before, HSPGs can be hybrid molecules, carrying both HS and CS side chains [15]. However, the alterations observed in transcriptions of GTs seem to point to changes in the CS chains. In addition, changes in CSPGs associated with breast cancer have been described, such as decorin or CSPG4 [39,40]. CS/DS chain modifications involve 4-sulfotransferases that differentiate between CS and DS (Figure 10C) [40,41]: CHST3 and CHST7 are chondroitin 6-sulfotransferases involved in early modification of the chain and CHST15 transfers sulfate to the C6 of an already 4-O sulfated GalNAc residue. Epimerization of GlcA residues is catalysed by the product of the *DSE* gene, and its C2 position can be sulfated by an uronyl-2-sulfotransferase encoded by *UST*[40]. Analysis of differential transcription of CS chain modification genes in this work showed that this group undergoes further alterations: 4 out of 9 genes displayed relevant reduced relative abundance of mRNA. The changes did not affect epimerization, but did affect sulfation in all positions, in both metastatic and non-metastatic tumors. *UST* transcription was downregulated about 4 fold, which should reduce the sulfation at C2 of IdoA. C6 sulfation of GalNAc appeared to be greatly diminished since the two genes involved, *CHST3* and *CHST7*, were deregulated, on average, 8 fold. Finally, sulfation of C4 was also affected, as evidenced by the fact that *CHS12*, one of the sulfotransferases with higher levels of transcription, appeared downregulatred about 3 fold in non-metastatic and nearly 10 fold in the metastatic IDCs. Interestingly, *CHST11*, which has been recently reported as highly expressed in aggressive breast cancer cells and to be significantly lower in less aggressive cancer lines [40], did not show significant differences in this study.

HPSE is an endo-β-D-glucuronidase that cleaves specific β-D-glucouronosyl-N-acetyl-glucosaminyl linkages [42]. Its expression is induced in all major types of human cancer, and is often associated with reduced patient survival, increased tumor metastasis and higher microvessel density [20]. Here, analysis of *HPSE* transcripts in IDCs produced no significant differences despite the use of two different pairs of probes located in regions at a considerable distance from each other.

Alternative splicing has been reported to occur in the non-coding regions of HPSE [20], and there is a possibility that this could influence the levels of protein expression. Two mRNA species that display the same ORF but differ in the structure of their 5′ end, HPSE1a and HPSE1b, have been described [19]. In this study, qRT-PCRanalysis indicated that the HPSE1a isoform was expressed in both healthy and tumor tissues, and at levels comparable to those determined in the coding region of *HPSE*. In contrast, HPSE1b was translated at very low or undetectable levels.

Recently, regulatory elements have been described in the 3′ unstranslated region (UTR) of the HPSE gene [21]. A 185-bp sequence that mediates *HPSE* down-regulation has been identified, which includes an adenine/uracil-rich consensus element able to cause mRNA degradation. Transcripts for both forms were detected in our study, although shorter mRNA was the most abundant. The differences observed between tumors and healthy tissues were not sufficient to reach significance, although when the analysis was carried out considering the ratio of the relative expression levels of both forms (long form / short form) however, the results showed statistically significant values for metastatic tumors. This would appear to indicate a shift towards a more stable form of mRNA in this tumor type, which may affect the overexpression of the protein by increasing its translation without an increase in its transcription levels.

HPSE2 is a homologue of HPSE that lacks HS-degrading activity, although it is able to interact with HS with high affinity [20]. There are several proteins generated by alternative splicing, although only the wild-type, HPSE2c, is secreted and binds with HS with very high affinity, competing with HPSE [43]. HPSE2 is capable of associating with HPSE and thus possibly modulates the latter’s enzymatic activity and signaling properties. This has resulted in an anti-metastatic feature being proposed [20,43]. Analysis of *HPSE2* transcription in tumors showed a noticeable decline, 100% of the tumors being affected, both metastatic and non metastatic. The downregulation was aproximatly 30 fold, and was detected using probes common to all isoforms as well as probes against exons 3 and 4, which can selectively detect HPSE2c. These data were confirmed by immunohistochemical studies where tissue arrays showed a noticeable decrease in inmunoreactivity for all tumors analysed.

## Conclusions

Analysis of the differential expression of the genes involved in the biosynthesis of HSPGs in tumors indicated that about 25% experienced significant changes in their transcript levels. Although some variations were detectable only in non metastatic tumors, most were identified in both IDCs although several genes showed more intense changes in metastatic tumors than in non-metastatic, including PRCAN, CSGALNACT2, HS3ST4 and CHST12. The overexpression of HPSE in metastatic tumors has been widely referenced in the literature, although our results show that it did not appear to undergo changes in levels of transcription, although its protein levels could well be controlled by a more stable mRNA isoform. In contrast, HPSE2, a homologue of HPSE that is able to interact with HS with high affinity, hence it being proposed to have anti-cancer properties, exhibited strong underexpression in all the patients studied, pointing to this gene having an important role in breast tumor progression.

## Abbreviations

CS: Chondroitin sulfate; ECM: Extracellular matrix; GAG: Glycosaminoglycan; GalNAc: N-acetyl-D-galactosamine; GlcA: D-glucuronic acid; GlcNAc: N-acetyl-D-glucosamine; GT: Glycosyltransferase; HS: Heparan sulfate; HSPG: Heparan sulfate proteoglycan; IDC: Invasive ductal carcinoma; IdoA: Iduronic acid; NK: Natural Killer; PG: Proteoglycan; PBS: Phosphate buffered saline; rER: Relative expression ratio; TBS: Tris-buffered saline.

## Competing interests

The authors declare that they have not competing interests.

## Authors’ contributions

IF performed the qPCR experiments and data analyses and contributed to the histochemistry. OG carried out the CISH and immunohistochemistry. AC and SC contributed to sample preparation and data analyses. PM and AA provided technical support and criticaly reviewed the manuscript. LQ performed the co-ordination of the study and drafted the manuscript. All authors read and approved the final manuscript.

## Pre-publication history

The pre-publication history for this paper can be accessed here:

http://www.biomedcentral.com/1471-2407/13/24/prepub

## Supplementary Material

Additional file 1 qRT-PCR primer sequences. Click here for file
