# Supplementary material for: Specific genes involved in synthesis and editing of heparan sulfate proteoglycans show altered expression patterns in breast cancer
Source: BMC Cancer. 2013 Jan 17;13:24. doi: 10.1186/1471-2407-13-24 (PMC3561094; doi:10.1186/1471-2407-13-24)
Supplement: Additional file 1 — qRT-PCR primer sequences. [file 1471-2407-13-24-S1.pdf]

Additional file 1

Table S1: qRT-PCR primer sequences

**Gene**	**Gene ID**	**Primer sequence**
SDC1	6382	F 5’- CTCAGGTGCAGGTGCTTTG<br>R 5’- CTGCGTGTCCTTCCAAGTG
SDC2	6383	F 5’- GATGACGATGACTACGCTTCTG<br>R 5’- TGGAAGTGGTCGAGATGTTG
SDC3	9672	F 5’- CTCCTTTCCCGATGATGAAC<br>R 5’- CGACTCCTGCTCGAAGTAGC
SDC4	6385	F 5’- GGCAGGAATCTGATGACTTTG<br>R 5’- TCTAGAGGCACCAAGGGATG
GPC1	2817	F 5’- CATCGGGTGTGGAGAGTG<br>R 5’- TGAGCGTGTCCCTGTTGTC
GPC2	221914	F 5’- CTGGGACACGACCTGGAC<br>R 5’- GCCATCCAGTCATCTGCATAC
GPC3	2719	F 5’- CTGCTTCAGTCTGCAAGTATGG<br>R 5’- GTGGAGTCAGGCTTGGGTAG
GPC4	2239	F 5’- AGTGTGGTCAGCGAACAGTG<br>R 5’- CAAACATATCATTCAGGGATTTCTC
GPC5	2262	F 5’- GCCGCCCTGTAAGAACAC<br>R 5’- TCATTCCATGCTTCTCTTTGC
GPC6	10082	F 5’- CCAGGCATAAGAAATTTGACG<br>R 5’- CATGTACAGCATGCCATAGGTC
PRCAN	3339	F 5’- TGGACACATTCGTACCTTTCTG<br>R 5’- CACTGCCCAGGTCGTCTC
AGRN	375790	F 5’- ACTGTGTCTGCCCGATGC<br>R 5’- GACACTCGTTGCCGTATGTG
COL18A1	80781	F 5’- GTACAAGGGAGAGATTGGCTTTC<br>R 5’- TTTCTCTCCTTTCAATCCGTTC
B3GAT3	26229	F 5’- GAAGAACGTGTTTCTCGCCTAC<br>R 5’- CCTCAGATCCTTCTGCCGTA
EXTL2	2135	F 5’- TGAACTGGAAACCAATGCAG<br>R 5’- AGGAAATTGCTGCCAAACTG
EXT1	2131	F 5’- GAGACAATGATGGGACAGACTTC<br>R 5’- CTCTGTCGCTGGGCAAAG
EXT2	2132	F 5’- CTGGGACCATGAGATGAATA<br>R 5’- GATATCCCCAGGCATTTTGTA
CSGALNACT1	55790	F 5’- TCAGGGAGATGTGCATTGAG<br>R 5’- AGTTGGCAGCTTTGGAAGTG
CHSY1	22856	F 5’- GCCCAGAAATACCTGCAGAC<br>R 5’- GCACTACTGGAATTGGTACAGATG
CHPF	79586	F 5’- GGTGCACTATAGCCATCTGGA<br>R 5’- GGCACTTCGGAAATGAGG
CHSY3	337876	F 5’- GACTCAGTGTGTCTGGTCTTACG<br>R 5’- TTGCTATTGTGAAGGTCTTGGA
NDST1	3340	F 5’- CTGCCCTCTACCTGTTCCTG<br>R 5’- AACTGGATCTCCTCAAAGGTCTC
NDST2	8509	F 5’- CAAGAGCTGCGTACCAACC<br>R 5’- GAGGGTCCGTGTGTAGTTCAG

NDST3	9348	F 5'- CCTTGCAGAAGAGATGTTTGG<br>R 5'- GTAGCAGGATCAGTTCTTAGTTGTTG
NDST4	64579	F 5'- GACATTGGGCTCCATCTGAC<br>R 5'- GCTGCTGTCCATCAATAATTAGC
GLCE	26035	F 5'- TGTGGAAGTCCGAGACAGAG<br>R 5'- CTGGATTGGATAGAAATAGCCTTG
HS2ST1	9653	F 5'- TGGAGATGATTATAGACCAGGGTTAC<br>R 5'- GCTATGGCCACAGAAGAACG
HS6ST1	9394	F 5'- GCAGGGAGTGGAGCTAACAG<br>R 5'- AACAGTTCCAGTTCCCGAAA
HS6ST2	90161	F 5'- CGGTGCGATCTTCTCCAA<br>R 5'- AGGACGATCACGGCAAATAG
HS6ST3	266722	F 5'- CAACCACAGCCACACCAG<br>R 5'- CTTCTTCCATCACACATATGAAGAG
HS3ST1	9957	F 5'- CAGCCAGATGCCCTTCTC<br>R 5'- AGACTCGCTCAGGCACTTTG
HS3ST2	9956	F 5'- GATTGGTACAGGAGCCTGATG<br>R 5'- GGAGCCTCTTGAGTGACAAAG
HS3ST3 (*)	9955<br>9953	F 5'- TGGTACCGGGACCTGATG<br>R 5'- CGCGTGACGAAGTAACTGG
HS3ST4	9951	F 5'- TAGAGCCGCACTTCTTCGAC<br>R 5'- GGTTATTTGCCCATCCAAAG
HS3ST5	222537	F 5'- CATCCGGCAGTAGTCAAAGC<br>R 5'- TTGTGATTTGCTGAGGGTAGG
HS3ST6	64711	F 5'- GCCCTGCTGGAGTTTCTG<br>R 5'- GCGCTCGTAGCACCTGTC
SULF1	23213	F 5'- CCAGCAGAAGCCAAAGAAAG<br>R 5'- GAACGTGTCTGCCGAGTATG
SULF2	55959	F 5'- GCCTGCAAGAGAAGGACAAG<br>R 5'- AGCAGCTTGCGGAGTTTC
CHST11	50515	F 5'- CGCTGCTGGAAGTGATGA<br>R 5'- AGGATAAAGGATCCCAAGCAA
CHST12	55501	F 5'- GTAGCCGACAAATCCTTCCA<br>R 5'- ACCGGTTTACCTCTGACTTGAC
CHST13	166012	F 5'- CCGGCATTTGGAAACAGA<br>R 5'- TCCAGGTCATAGAGCTTCTGC
CHST14	113189	F 5'- CCACTGCCTAATGTCACCAA<br>R 5'- ATGACAGGCAGAAGCACAGA
CHST15	51363	F 5'- GTGCCAGGAATAAAGTTCAACA<br>R 5'- CACTGGATAAGTCCCGAGTGA
CHST3	9469	F 5'- TGCACAGCCTGAAGATGAGA<br>R 5'- CAGCTTGTCTGAGACCCTTGA
CHST7	56548	F 5'- GATCCGGGTCAGTCACCA<br>R 5'- GACAGATTGCCCCCACAG
DSE	29940	F 5'- GTCCAGAGGCACTTCAACATC<br>R 5'- AGTCCGCAATAGCCACAGTC
UST	10090	F 5'- ACCATGGACCACCTCCTAGTAA<br>R 5'- CACACTTGCCTACCCTGTTGTA
HPSE (exons 5-6)	10855	F 5'- ATGCTCAGTTGCTCCTGGAC<br>R 5'- CTCCTAACTGCGACCCATTG
HPSE (exons 9-10)	10855	F 5'- CCTTGCTATCCGACACCTTTG<br>R 5'- TATTCTTTGGAGCAGGAAACTACC
HPSE 1a	10855	F 5'- GCGGGAGGAAGTGCTAGA<br>R 5'- CCAGGAGCAGCAGCATCA

HPSE 1b	10855	F 5'- TCTCCGCACCCTTCAAGT<br>R 5'- CCAGGAGCAGCAGCATCA
HPSE (full 3' UTR)	10855	F 5'- AATGCCAAAGTTGCTGCTTG<br>R 5'- GCGAGATCAAAATACTGTGCTAAA
HPSE (short 3' UTR)	10855	F 5'- CAGGAAGTTCACTGGGCTTG<br>R 5'- CGTCACCCACTAGTTGCTTTG
HPSE2c	60495	F 5'- CACCCTGATGTTATGCTGGAG<br>R 5'- TCCAGAGCAATCAGCAAAGTTA
HPSE2	60495	F 5'- GGCCGAGGAAGAATGTCA<br>R 5'- GTGTGTCTAACAGGCGAGTTTTC

(*) This pair of primers identifyes a sequence that is common to HS3ST3A1 and HS3ST3B1
